# Use of artificial intelligence to measure colorectal polyp size without a reference object

**DOI:** 10.1055/a-2556-1836

**Published:** 2025-05-12

**Authors:** Chin-Yuan Yii, Ding-Ek Toh, Tzu-An Chen, Wei-Lun Hsu, Huang-Jen Lai, Yin-Chen Wang, Chang-Ru Liu, Yow-Chii Kuo, Shih-Hao Young, Fu-Ming Chang, Chen Lin

**Affiliations:** 1 215168 Division of Gastroenterology and Hepatology, Department of Internal Medicine, Landseed International Medical Group, Taoyuan, Taiwan; 2 34911 Department of Biomedical Sciences and Engineering, National Central University, Zhongli, Taiwan; 3 14351 Department of Gastroenterology, Flinders Medical Centre, Bedford Park, Australia; 4 63474 Division of Gastroenterology and Hepatology, Department of Internal Medicine, Taipei Medical University Hospital, Taipei, Taiwan; 5 215168 Division of Colorectal Surgery, Department of Surgery, Landseed International Medical Group, Taoyuan, Taiwan; 6 34911 Institute of Computer Science and Information Engineering, National Central University, Zhongli, Taiwan; 7 215168 Nursing Department, Landseed International Medical Group, Taoyuan, Taiwan

**Keywords:** Endoscopy Lower GI Tract, Polyps / adenomas / ..., CRC screening, Colorectal cancer

## Abstract

**Background and study aims:**

Polyp size is crucial for determining colonoscopy surveillance intervals. We present an artificial intelligence (AI) model for colorectal polyp size measurement without a reference object.

**Methods:**

The regression model for polyp size estimation was developed using outputs from two SegFormer models, segmentation and depth estimation. Initially built on colonoscopic images of polyp phantoms, the model underwent transfer learning with 1,304 real-world images. Testing was conducted on 178 images from 52 polyps, independent of the training set, using a snare as the ground truth for size comparison with the AI-based model. Polyps were classified into three size groups: ≤ 5 mm, 5–10 mm, and ≥ 10 mm. Error rates were calculated to evaluate discrepancies in actual size values between the AI model and the snare method. Precision indicated the positive predictive value per size group and recall and Bland-Altman were also conducted.

**Results:**

The Bland-Altman analysis showed a mean bias of –0.03 mm between methods, with limits of agreement from –1.654 mm to 1.596 mm. AI model error rates for actual size discrepancies were 10.74%, 12.36%, and 9.89% for the ≤ 5 mm, 5–10 mm, and ≥ 10 mm groups, respectively, averaging 11.47%. Precision values were 0.870, 0.911, and 0.857, with overall recall of 0.846.

**Conclusions:**

Our study shows that colorectal polyp size measurement by AI model is practical and clinically useful, exhibiting low error rates and high precision. AI shows promise as an accurate tool for measurement without the need for a reference object during screening colonoscopy.

## Introduction

Colorectal cancer (CRC) stands as one of the most prevalent malignancies globally. Implementation of CRC screening programs may reduce CRC-related mortality 1 . CRC has been the most common cancer for 15 years consecutively in Taiwan. According to statistics from the Taiwanese Ministry of Health and Welfare, it was the most common cancer in males and the third most common in females in 2020 2 . The age-standardized mortality rate for CRC has increased and it was the third leading cause of cancer deaths, with 6,853 deaths in 2022.

Current knowledge recognizes three distinct pathways through which CRC can emerge: the adenoma-carcinoma sequence, the serrated pathway, and the inflammatory pathway 3 . The majority of cases are sporadic, influenced by factors such as dietary lifestyle, polyp size, polyp number and presence of dysplasia.

According to current guidelines from the European Society of Gastrointestinal Endoscopy (ESGE) and American Society for Gastrointestinal Endoscopy, clinical decision-making regarding CRC treatment modalities is heavily influenced by polyp size and characteristics of invasiveness 4 5 6 . However, visual estimation of polyp size remains high variable among endoscopists, even those with extensive experience 7 . In light of advancements in artificial intelligence (AI) technology, AI techniques for polyp detection and characterization are becoming a reality, as well as the exciting prospect of reliable AI-technique polyp size measurement.

Presently, Fujifilm Europe 8 and Argus, powered by EndoSoft 9 , have introduced software with size estimation capabilities. However, both solutions require a reference object for measurement. Addressing this, EndoAim 10 , developed by ASUSTeK Computer Inc., is now one of the AI systems for colonic polyp detection and characterization. Our objective was to enhance EndoAim by incorporating a new application for polyp size measurement without need for a reference object. This enhancement involves installing verified images or video into EndoAim to generate accurate size measurement.

We aimed to develop an AI model for estimating colorectal polyp size without the need for a reference object, intended for use in screening colonoscopy.

## Methods

### Construction of preliminary AI system and development of model for polyp size estimation

**Fig. 1:**
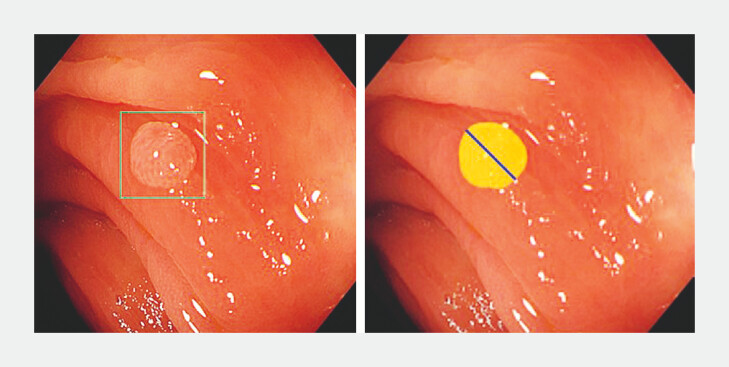
Colorectal polyp with bounding box (left) and its segmentation (right). The blue line represents two endpoints of longest axis.

**Fig. 2:**
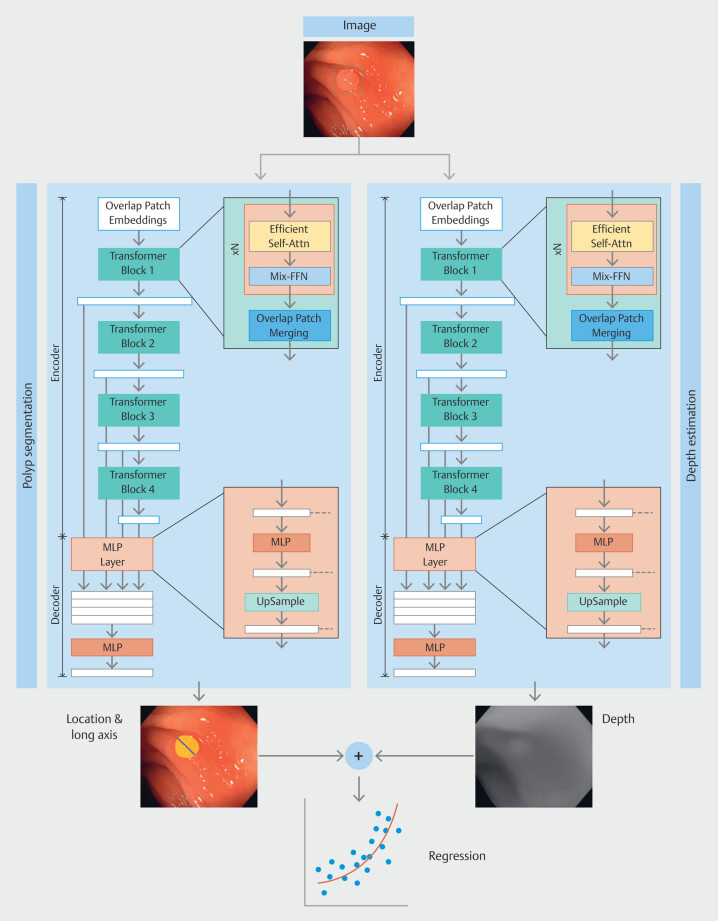
Proposed incorporated framework of the model.

Image segmentation is a computer vision task, by producing a dense pixel-wise segmentation map of an image. Each pixel is then dedicated to a specific class or object. We used two open datasets, bkai-igh-neopolyp and kvasir-seg, for segmentation model training. For this technique, a polyp was outlined from a colonoscopy image and sketches were made of the two endpoints of its longest axis ( Fig. 1 ). Still figures with image segmentation were captured from a dynamic video colonoscopy. For real-time image segmentation, we employed SegFormer, a robust and efficient approach for semantic segmentation, to develop two independent models for monocular depth estimation 11 and polyp detection. SegFormer integrates a hierarchical transformer encoder with a lightweight multilayer perceptron (MLP) decoder, delivering both simplicity and computational efficiency while maintaining top-tier performance across various benchmarks. As shown in Fig. 2 , the framework processes images cropped and resized to 720 × 576 pixels, which are then used to train two separate SegFormer models: one for depth estimation and the other for polyp segmentation. Output features from both models are concatenated to form a regression model for estimating polyp size.

### Snare as a tool for ground truth

**Fig. 3:**
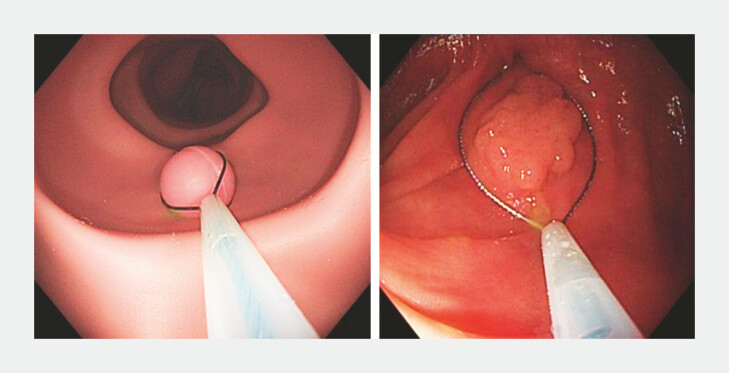
Schematic of an in vitro polyp with a fully opened snare (left) and a polyp surrounded by a 15-mm Boston Captivator II snare (right).

**Fig. 4:**
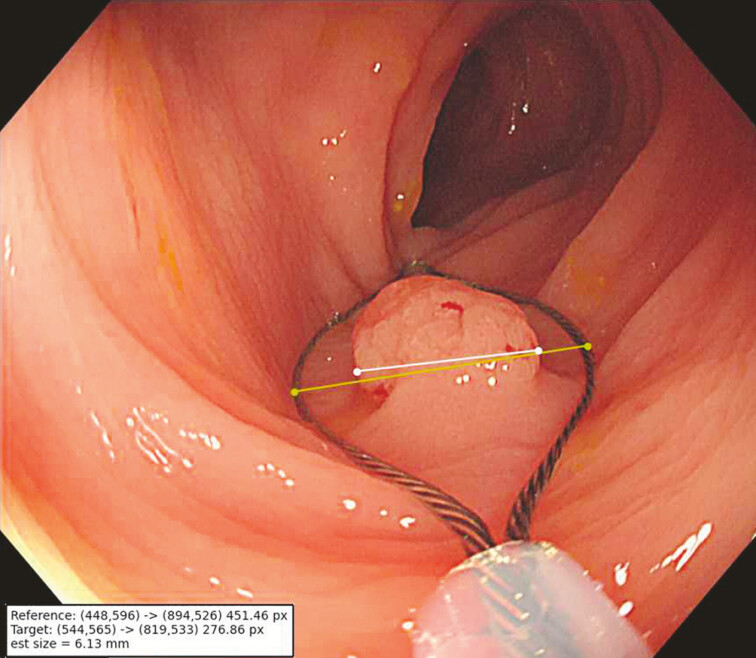
The yellow line represents the maximum length of both snare edges, 10 mm; the white line indicates digital size of the polyp, 6.13 mm.

**Fig. 5:**
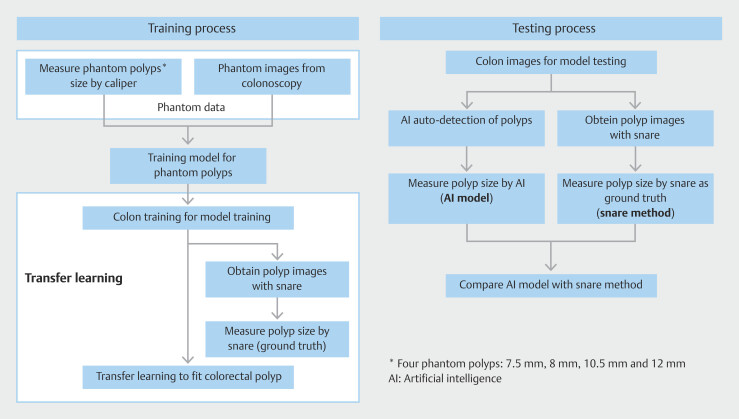
Flowchart of training and testing process.

The ground truth of this model is based on the fully opened snare, which was not deformed. Clear images of the polyp within the snare ( Fig. 3 ) were selected. Both snare edges should be visualized for measurement. A Boston Captivator II single-use snare was chosen in the study, either 10 mm or 15 mm, due to its stiffness. The model was trained with a learning rate of 0.001 over 60,000 iterations. Actual polyp size was calculated in millimeters by contrasting the two coordinates – maximum snare edges and the polyp – with the former serving as a standard reference ( Fig. 4 ). In our study, the AI model was used to measure polyp size during routine colonoscopies without any reference object. The development flowchart, including the training and testing processes, is depicted in Fig. 5 .

### AI system construction

#### Model training

**Fig. 6:**
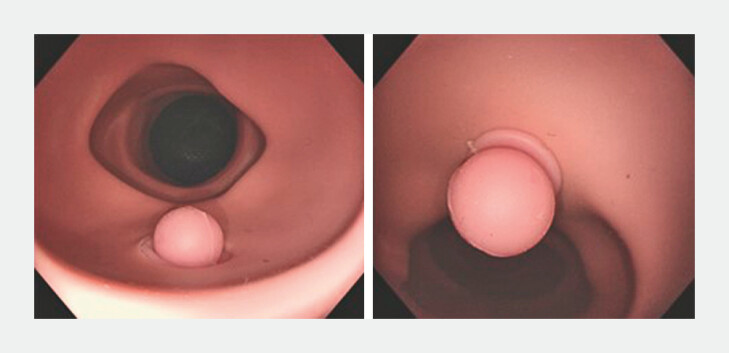
Polyp phantoms.

The initial model was trained on four detachable polyp phantoms measuring 7.5 mm, 8 mm, 10.5 mm, and 12 mm ( Fig. 6 ). Size estimation was performed according to the aforementioned system. Because polyp phantoms are not representative of real polyps, transfer learning was conducted. From May to July, 2022, 172 colon polyps from 50 patients in Landseed International hospital were retrospectively reviewed. A total of 1304 images were used for learning. The study was approved by the Ethics Committee of Landseed International hospital (approval number: IRB-23-063-C0).

#### Model testing

**Fig. 7:**
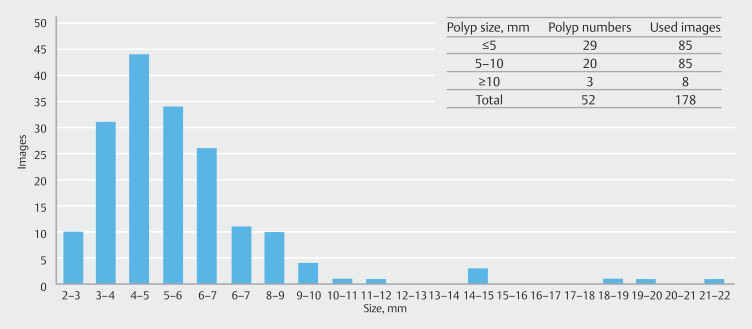
Fifty-two polyps were selected with 178 usable images for validation.

From May to October, 2023, 52 polyps were collected retrospectively from 257 routine colonoscopies by a single endoscopist, Yii CY. In total, 178 images with polyps centered by a snare were selected for validation ( Fig. 7 ). The study was approved by the Ethics Committee of Landseed International hospital (approval number: IRB-23-055-C0). An Olympus 290 series colonoscope, CF-H290L/I, was used in this model.

**Fig. 8:**
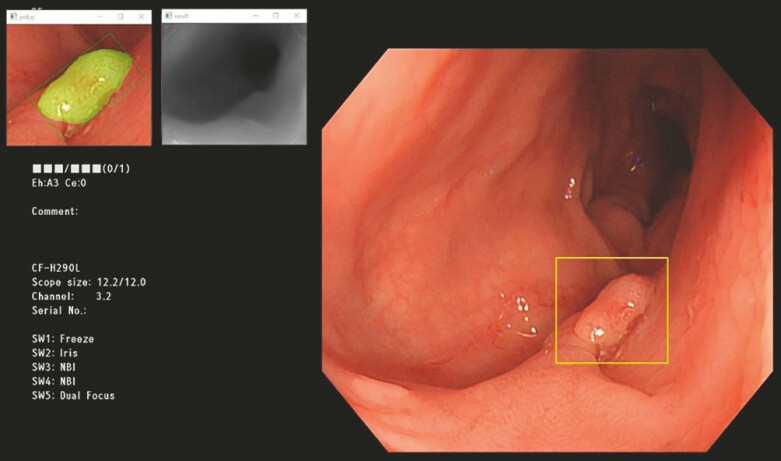
AI model of auto-detection, segmentation, depth estimation, and automated size estimation.

Polyp size obtained by snare was then compared with auto-estimation of size by the AI model. Fig. 8 shows the process of AI auto-estimation.

#### Colonoscope

An Olympus 290 series colonoscope with s 170° field of view (FOV) was used, such as CF-H290L/I, CF-HQ290L/I, CF-HQ290ZL/I, PCF-H290L/I, PCF-H290DL/I, PCF-H290ZL/I. In addition, optical or digital zoom and near focus function of the colonoscope were not allowed.

#### Statistical analysis

Polyps were allocated to three size groups: ≤ 5 mm, 5 to 10 mm, and ≥ 10 mm. Error rates represented the size difference between the snare method and the AI model. It was calculated by the following formula, $\frac{\left(size\;estimated\;by\;snare\;method\;-\;size\;estimated\;by\;AI\;method\right)}{size\;estimated\;by\;snare\;method}\times 100\%$ . Each of the three size groups was calculated separately. Precision indicated the proportion of predicted true positive rate, comparing the snare method and the AI model, in each of the three size groups. Estimated polyp sizes, as determined by both the AI model and the snare method, were summarized using means and standard deviations. To assess the size difference between these two methods, a Bland-Altman plot was constructed. In addition, sizes measured by the AI model and the snare method were statistically compared using a paired *t* -test. Polyp sizes were categorized into three groups (≤ 5 mm, 5–10 mm, and ≥ 10 mm). Cohen's Kappa coefficient was calculated to evaluate agreement between the two methods for these categorical size ranges. All statistical analyses were performed using R software, version 4.2.1. A *P* < 0.05 was considered to indicate statistical significance.

#### Sample size estimation

Sample size for our study was determined through a power analysis. This analysis utilized the mean and standard deviation of the differences between the size estimates in the phantom obtained by the AI model and the snare method, or absolute percentages of 7% and 10%, respectively. The analysis aimed for a significance level (alpha) of 0.05 and a power of 90% in a two-tailed paired *t* -test. This approach calculated the minimum required sample size to ensure at least 20 polyps.

## Results

### Performance for polyp measurement

Table 1 shows that errors rate for polyps ≤ 5 mm, 5 to 10 mm, and ≥ 10 mm were 11.36%, 11.46%, and 23.24%, respectively, for the training model, and 10.74%, 12.36%, and 9.89%, respectively, for the testing model. The average error rate was 11.80% (95% confidence interval [CI] 10.29–13.32) for the training model, and 11.47% (95% CI 10.27–12.68) for the testing model.

**Table:** **Table 1** Error rates for the snare method and AI model for three polyp size ranges: ≤ 5 mm; 5 to 10 mm, and ≥ 10mm.

**Training model**				
Polyp size (mm)	≤ 5 mm	5–10 mm	≥ 10 mm	Overall
Error (%) (95% CI)	11.36 (8.75–13.97)	11.46 (9.70–13.23)	23.24 (8.52–37.96)	11.80 (10.29–13.32)
**Testing model**				
Polyp size (mm)	≤ 5 mm	5–10 mm	≥ 10 mm	Overall
Error (%) (95% CI)	10.74 (9.23–12.24)	12.36 (10.41–14.32)	9.89 (4.33–15.44)	11.47 (10.27–12.68)
CI, confidence interval of standard error of the mean.				

### Variation in accuracy of size classifications

If a predicted polyp size by the AI method and the snare method were within the same size group, it was considered as true. As shown in Table 2 , the precision for polyps ≤ 5 mm, 5 to 10 mm, and ≥ 10 mm was 0.870, 0.911, and 0.857, respectively, with an average of 0.879 in the testing group. Recall in the testing group represented size measurements that were correctly predicted by the AI model for the snare method. Recall for polyps ≤ 5 mm, 5 to 10 mm, and ≥ 10 mm was 0.941, 0.847, and 0.750, respectively, with an average of 0.846. Calculating the harmonic mean of the precision and recall, the overall F _1_ score was 0.861.

**Table:** **Table 2** Precision, recall, and F _1_ score of testing model in three polyps size groups: ≤ 5 mm; 5–10 mm, and ≥ 10 mm

Snare method AI model	≤ 5 mm	5–10 mm	≥ 10 mm	All
≤ 5 mm	80	12	0	92
5–10 mm	5	72	2	79
≥ 10 mm	0	1	6	7
All	85	85	8	178
Precision	0.87	0.911	0.857	0.879
Recall	0.941	0.847	0.75	0.846
F _1_ Score	0.904	0.878	0.8	0.861

### Variability in size measurement between the AI model and the snare

**Fig. 9:**
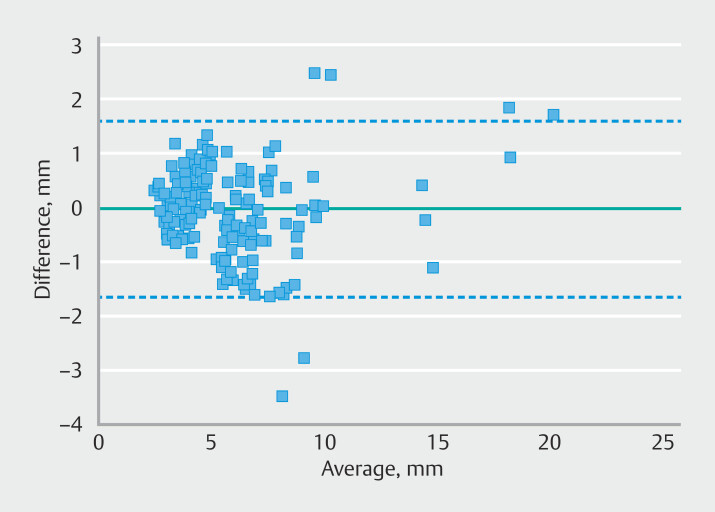
Bland-Altman plot showed that there were six cases (3.4%) out of range of the 95% confidence interval, ± 1.6 mm (the green dashed line), whereas 33 cases (18.5%) exceeded ±1 mm (the blue dashed lines).

Cohenʼs kappa value for the comparison between the snare and the AI model across the three polyp size groups was 0.792, indicating strong consistency between these two distinct approaches. Moreover, the Bland-Altman analysis demonstrated a mean bias of –0.03 mm between the methods, with no significant differences observed in the paired *t* -test ( *P* = 0.638). Limits of agreement were calculated as mean difference ± 1.96 standard deviations of the differences, ranging from –1.654 mm to 1.596 mm, as depicted in Fig. 9 . Of 178 analyzed images, six images (3.4%) fell outside the 95% agreement limits, with a maximum absolute difference of 3.47 mm.

## Discussion

In this study, we developed a novel AI model to measure colon polyp size without need for a reference object. Initially trained on colonoscopic images of polyp phantoms, the model underwent transfer learning with real-world data. We systematically compared AI-based model performance against the traditional snare method, which served as the ground truth in our testing dataset. This AI system has the potential to accurately measure colorectal polyp size and assess polyp characteristics, aiding in selection of appropriate polypectomy techniques and determining surveillance intervals, ultimately contributing to reduced CRC risk.

A European study by Erlangen Group 12 proved that adenoma size was the most important factor related to invasive carcinoma in a database of 11,188 adenomas from 1978 to 1993. A total of 5027 adenomas (44.9%) that were less than 5 mm carried no cancer risk. In addition, the cancer risk increased proportionate with adenoma size, especially for right-sided colon adenomas 12 13 . Polyp size is one of the major determinants of the resection plan and interval for surveillance 13 14 15 . Recent ESGE guidelines 16 emphasize different and appropriate polypectomy techniques in relation to polyp characteristics and size. For instance, cold snare polypectomy is recommended for polyps less than 9 mm, hot snare polypectomy for polyps 10 to 19 mm, and endoscopic mucosal resection for polyps larger than 20 mm.

Given the high volume of colonoscopy workload, a few studies have suggested a “discard” strategy for polyps ≤ 5 mm without presence of unfavorable features endoscopically 17 18 . The 5-mm threshold is crucial to this “resect and discard” strategy and aids endoscopist decision about cold or hot polypectomy 19 . Some studies set polyp size ≥ 10 mm as a cut-off reference because of the potential cancer risk 20 . Although advanced histology has been identified, frequent follow-up is recommended in these cases 4 14 . The number of adenomas also dictates the surveillance interval. According to the American guideline, patients with only one or two low-grade dysplastic tubular adenomas ≤ 10 mm should have the next follow-up in 5 to 10 years; those with three to 10 adenomas, or any adenoma > 10 mm, or any adenoma with high-grade dysplasia or villous histology should have follow-up in 3 years 21 . Polyps less than 10 mm may be resected immediately during routine colonoscopy 22 23 . Polyps that are ≤ 20 mm need to be evaluated for immediate snare with endoscopic mucosal resection or scheduled endoscopic submucosal dissection 24 . It is hoped that polyp size measurement with AI can allow the examiner to decide on the spot whether to remove a polyp or leave it, making the examination smoother and faster.

Categorization of polyp size into ≤ 5 mm, 6 to 9 mm, and ≥ 10 mm is widely recognized as clinically important, as supported by several studies 17 18 25 26 . In our study, we grouped polyps into three different size categories: ≤ 5 mm, 5 to 10 mm, and ≥ 10 mm, aiming for model precision of one to two decimal places, especially for smaller polyps. Our observations revealed that four of six images exceeded the CI, likely due to factors such as surrounding feces, orientation, distance, and direction of the open snare, as shown in Supplementary Fig. 1. In addition, we noted that larger snares could cause compression and deformation, introducing measurement errors. Discrepancies between the AI-based model and snare measurements were more pronounced for larger polyps. Specifically, two of the six images (Supplementary Fig. 2) depicted polyps approximately 20 mm in size, with discrepancies greater than 1.6 mm between the AI model and the snare method (1.72 mm and 1.85 mm, respectively). However, the error rate remained below 10% due to their large size. Furthermore, lateral spreading tumors, which span one to two colonic folds and are significantly large, also present challenges for accurate AI measurement. Given the infrequency of polyps exceeding 15 mm, the limited number of such images in our test model might also diminish confidence in measurements of larger polyps.

There are now two commercial AI products for polyp size measurement, Fujifilm Scale Eye and EndoSoft Argus, with laser and snare tip as reference objects, respectively. From the literature review, several tools were used as reference for size estimation, such as forceps 27 28 , ruler 7 , calibrated hood 29 , graduated injection needle 30 , or snare 26 . A study also has been reported of use of a novel system called Poseidon for measurement by using the auxiliary waterjet as ground truth 31 . Kwak MS et al. reported on an AI technique for measuring polyp size by bifurcation-to-bifurcation distance of colon vessels 32 . Until now, there was no ideal ground truth for polyp size measurement. Every tool encounters some bottlenecks and inaccuracy. In this study, we used the snare as ground truth because of its availability, convenience, and its common use in daily practice. We selected images in which the snares were not distorted or compressed. We used only the maximum width of the snare that was officially written on the product cover, such as 10 mm, as our ground truth.

To ensure consistency of snare-based polyp measurement across different physicians, we asked three physicians to independently measure the size of snare-based polyps using the same coordinate-based method. To assess agreement among them, Fleiss' kappa was used and a Single Score Intraclass Correlation (ICC) was calculated across 178 subjects. The resulting ICC was 0.942 (95% CI 0.923–0.956), demonstrating a very high level of consistency. In addition, the kappa value of 0.882 indicates almost perfect agreement ( *P* < 0.001). Both the ICC and Fleiss' kappa confirm that the three raters achieved a very high level of consistency in their measurements and categorizations.

Our study has several limitations that warrant acknowledgment. First, the snare was used as the ground truth for polyp size measurement. Future studies could benefit from incorporating additional measurement methods for comparison, such as direct measurement of the resected specimen. Second, having a single endoscopist in our testing model caused selection bias and could lead to poor generalizability of the model. Third, retrospective review of endoscopic pictures introduces a potential bias. Performance of a prospective study conducted in real-time colonoscopy would have enhanced the reliability of our findings. Fourth, the study would benefit from a larger sample size of polyps, ideally in a multicenter setting. This approach aims to mitigate potential biases associated with a limited number of cases. Fifth, this method is currently tailored only to an Olympus 290 series colonoscope with a 170° FOV. Extending the study to include a 140° FOV and exploring compatibility with other endoscope manufacturers is mandatory for a comprehensive understanding. Sixth, we used a Boston Captivator II snare as the sole reference tool, which may limit the generalizability of our results. Considering alternative reference tools, such as a hexagonal snare, on-site polyp measurement by caliper, snare tip, and forceps, would enhance the comparability and robustness of our study.

## Conclusions

In summary, our model demonstrated streamlined efficiency in screening colonoscopy through accurate polyp size measurement with no reference object required. This significant advancement aligns with increasing integration of AI in healthcare.

## Supplementary Material

Supplementary Material Zusatzmaterial
