# Supplementary material for: Use of artificial intelligence to measure colorectal polyp size without a reference object
Source: Endosc Int Open. 2025 May 12;13:a25561836. doi: 10.1055/a-2556-1836 (PMC12080523; doi:10.1055/a-2556-1836)

Robustness of the snare for ground truth

To assess the robustness of the snare as our ground truth, we conducted a comparative analysis of various snares from different manufacturers and ultimately selected the Boston Captivator II. This snare features a 1 x 7 braided design, which offers greater resistance to deformation. Our tests confirmed its maximum width is optimal when fully opened. We placed a 10-mm snare on a ruler, using the ruler scale at 2-mm intervals from 2 mm to 12 mm, as a reference standard (**Supplementary Table 1**). We then delineated the maximum width of the snare with a fixed 10-mm yellow line, and a white line was generated as an estimated scale using a coordinate-based comparison method. The ruler scale and the estimated scale showed high similarity, with differences less than 1 mm. In addition, we used a digital caliper to measure the maximum width of the snares when fully opened, with findings presented in **Supplementary Table 2**, showing how closely each snares width approximated 10 mm.

Endosc Int Open | DOI 10.1055/a-2556-1836 | © The Author(s).

Supplementary Table 1 Calculation of an estimated scale using a coordinate-based comparison method with a ruler scale as the reference.

	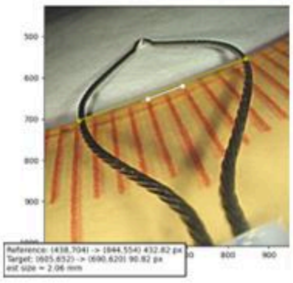	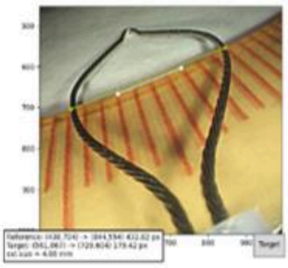
Ruler scale/ Estimated scale	2mm/2.06mm	4mm/4.06mm
	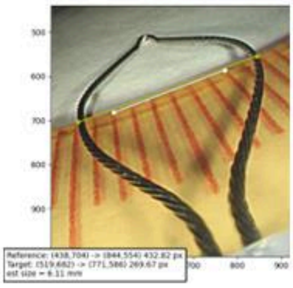	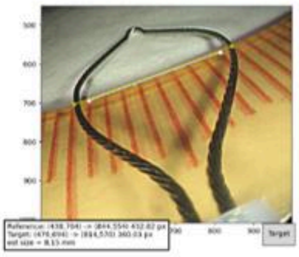
Ruler scale/ Estimated scale	6mm/6.11mm	8mm/8.15mm
	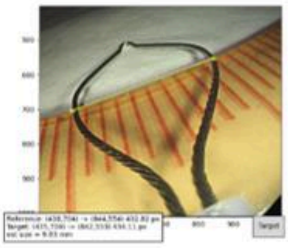	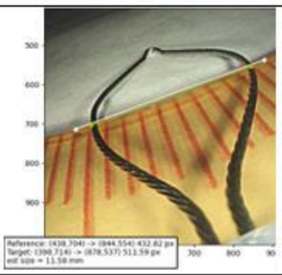
Ruler scale/ Estimated scale	10mm/9.83mm	12mm/11.58mm

Supplementary Table 2 Maximum width of the fully-open snares that were measured by digital caliper.

	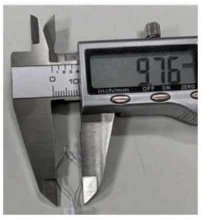	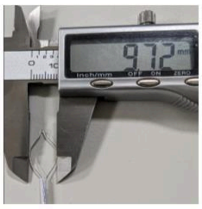	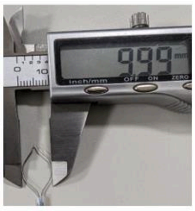	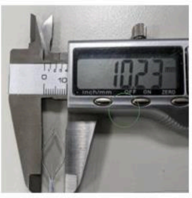	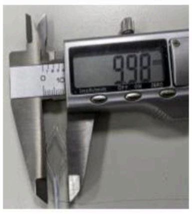
Snare	1	2	3	4	5
Caliper measurement	9.76mm	9.72mm	9.99mm	10.23mm	9.98mm
Mean±SD	9.82±0.27mm				

Supplementary figure 1: These two figures showed large differences between AI model and snare method, which were probably due to the surrounding interference (e.g feces), orientation, distance and direction of the open snare

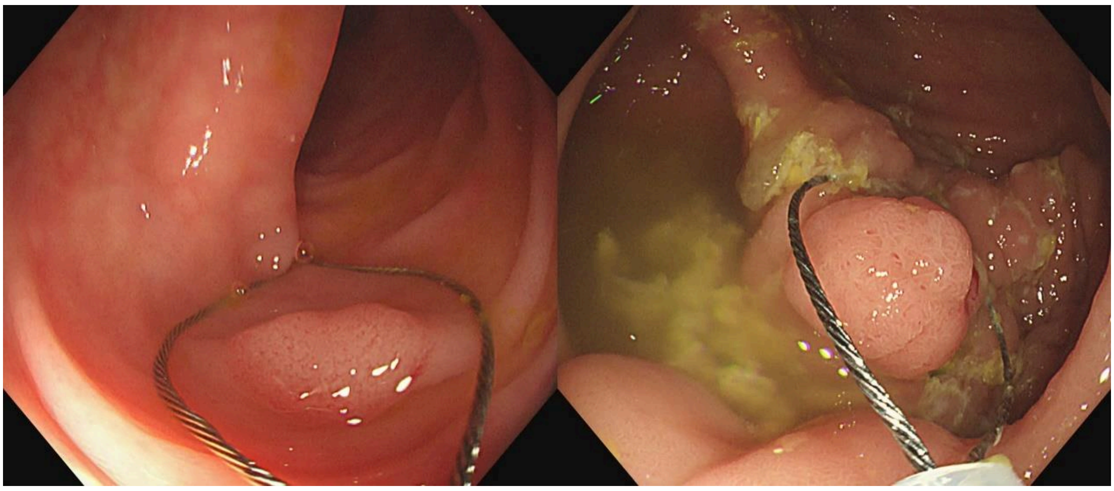

Supplementary figure 2: The two 20mm large polyps with difference of AI model and snare method greater than 1.6mm

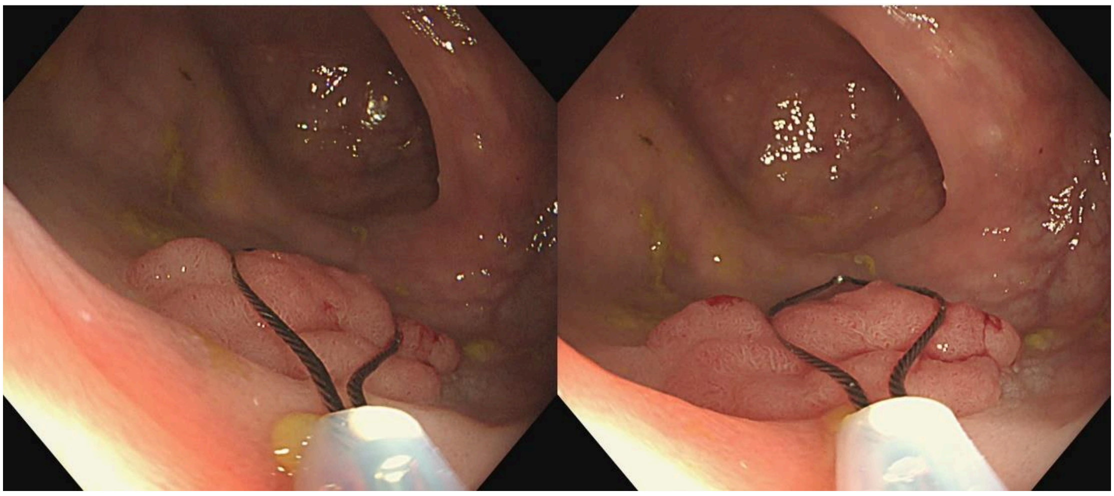

Supplement: Supplementary file 1 — Supplementary Material [file 10-1055-a-2556-1836_25562921.pdf]
